# Why study EU foreign policy at all? A response to Keuleers, Fonck and Keukeleire

**DOI:** 10.1177/0010836716682393

**Published:** 2016-12-14

**Authors:** Hylke Dijkstra, Sophie Vanhoonacker

**Keywords:** European Union, foreign policy, research agenda, theory

## Abstract

In an important article on the state of European Union (EU) foreign policy research, Keuleers, Fonck and Keukeleire show that academics excessively focus on the study of the EU foreign policy system and EU implementation rather than the consequences of EU foreign policy for recipient countries. While the article is empirical, based on a dataset of 451 published articles on EU foreign policy, the normative message is that it is time to stop ‘navel-gazing’ and pay more attention to those on the receiving end of EU foreign policy. We welcome this contribution, but wonder why certain research questions have been privileged over others. We argue that this has primarily to do with the predominant puzzles of the time. We also invite Keuleers, Fonck and Keukeleire to make a theoretical case for a research agenda with more attention to outside-in approaches. We conclude by briefly reflecting on future research agendas in EU foreign policy.

## Introduction

It is regularly observed that more academics study European Union (EU) foreign policy than there are civil servants to make it work.^1^ In their contribution to *Cooperation and Conflict*, Keuleers et al. (2016) now show that a large proportion of these academics primarily examine what those civil servants do.

Based on a dataset of 451 articles on EU foreign policy, published in key journals between 2010 and 2014, they identify three research approaches: first, the ‘inward-looking’ approach which focuses on the EU foreign policy system itself; second, the ‘inside-out’ approach which assesses the implementation of EU foreign policy; and third, the ‘outside-in’ approach which analyses the consequences of EU foreign policy for recipient countries. They find that academic work is ‘rife with EU-centric research questions’ (Keuleers et al., 2016: abstract) and that the outside-in approach accounts for less than 20 per cent of publications (Keuleers et al., 2016: figure 2).^2^ While their article is empirical, the normative message is that it is time to stop ‘navel-gazing’ (Keuleers et al., 2016: title) and pay more attention to those on the receiving end of EU foreign policy.

We are the (co-)authors of 8/192 articles classified in their dataset as inward-looking. Our purpose here is not to defend our own perspective. We would like to build on the findings of Keuleers et al. (2016) to discuss *why certain research questions have been privileged over others*. In other words, why is it that so many scholars have decided to focus on ‘agenda setting, policy formulation and decision-making’ (Keuleers et al., 2016: 348). We argue that this has primarily to do with the predominant puzzles of the time. Secondly, we invite Keuleers et al. (2016) to make a theoretical case for a research agenda with more attention for outside-in approaches. We conclude by briefly reflecting on future research agendas in EU foreign policy.

## Why all the navel-gazing?

Academic debates are generally driven by puzzles which inform research questions. To answer these questions, scholars develop and make use of theories. It is worth exploring what the puzzles and theories have been in EU foreign policy research. In order to understand research choices, particularly in the period 2010–2014, it is helpful to look at the broader scholarly debate since the 1960s.

In an article which reflects on ‘the end of International Relations theory’ Dunne et al. (2013: 412–413) identify three key drivers behind theoretical development and academic research. First, they note that new theories get invented ‘in light of a general perception on the part of the academic community that a new historical context requires new conceptual tools of analysis’ (Dunne et al., 2013: 412). Looking at the academic debate since the establishment of European Political Cooperation in 1970, it is indeed the case that EU foreign policy research cannot be understood independently from the international context. The discussions on Civilian Power Europe were prominent during a period of *détente* in the 1970s (Duchêne, 1973). The actorness debate was critical in the 1990s when the EU launched the Common Foreign and Security Policy and made an attempt to develop its own international voice (Allen and Smith, 1990; Jupille and Caporaso, 1998). The Normative Power Europe debate emerged when it became increasingly clear that the role of the EU’s crisis management role would mainly be civilian rather than military in nature (Manners, 2002).

A second driver for ‘theoretical proliferation’, identified by Dunne et al. (2013: 413), is ‘the practice of “importing” a theory from a cognate discipline’. Once again, EU foreign policy is no exception. In the early years of European foreign policy cooperation scholars used international relations theory to explain why foreign policy integration did not occur (Bull, 1982; Hoffmann, 1966; Waltz, 1979: 152). When it did appear that EU foreign policy was becoming increasingly significant scholars started using meso-level theories from cognate fields. By the late-1990s and early-2000s, Europeanisation and governance theories, building on institutionalist theories and imported from EU public policy, gradually found their way to foreign policy scholars (Dijkstra, 2008; Duke and Vanhoonacker, 2006; Juncos and Pomorska, 2006; Manners and Whitman, 2000; Smith, 2004; Tonra, 2001; Wong, 2005).

Finally, as Dunne et al. (2013: 413) note, ‘theoretical proliferation can be located in the developments within the discipline itself’. This also seems relevant for EU foreign policy. One only needs to point to the ‘Europe as a power’ debate. Following the keynote article by Manners (2002), scholars have proposed a wide range of adjectives: from realist to ethical and market power Europe (Aggestam, 2008; Damro, 2012; Hyde-Price, 2006). This has been a theoretical debate within the EU foreign policy research. The finding that the EU as a non-state actor has a degree of actorness has likewise triggered extensive theoretical debate (Bretherton and Vogler, 1999; Groenleer and Van Schaik, 2007; Hill, 1993; Jupille and Caporaso, 1998).

With these three drivers in mind, it should not come as a surprise that so many scholars have focused on institutional questions (inward-looking approach) and implementation questions (inside-out approach) during the 2010–2014 timeframe analysed by Keuleers et al. (2016). The Treaty of Lisbon of 2009 was a historic leap forward in terms of the EU-level diplomatic system with the High Representative for Foreign Affairs and Security Policy, the European External Action Service and the EU delegations. It posed a major research puzzle: never before did we witness such a centralisation of diplomatic resources in a non-state actor. It is thus hardly surprising that many scholars studied these new developments (Dijkstra, 2013; Juncos and Pomorska, 2013; Spence and Bátora, 2015; Vanhoonacker and Pomorska, 2013). The development of a European-level diplomatic system furthermore gave a new impulse to the use of concepts and insights of public administration (Henockl, 2014; Vanhoonacker et al., 2010).

During the 2010–2014 period scholars furthermore systematically tested – including through cross-case comparisons – theoretical approaches developed during earlier periods. Among others, at least two special issues of the *Journal of European Public Policy* and *International Relations*, were published on actorness with a view of driving this concept forward (Da Conceição-Heldt and Meunier, 2014; Niemann and Bretherthon, 2013). Normative power was also critically analysed in a special issue of *Cooperation and Conflict* (Nicolaïdis and Whitman, 2013). The period from 2010 to 2014 also included an authoritative volume on the Europeanisation of national foreign policy (Wong and Hill, 2011). In other words, while scholars generated theories of EU foreign policy during the 2000s, they tested them during the early-2010s.

## The academic relevance of foreign policy analysis

Our argument thus far has been that puzzles and theories drive academic research in the area of EU foreign policy. This helps us to explain why scholars have privileged inward-looking and inside-out approaches. The big question is how the outside-in perspectives fit in. In this section, we suggest that if Keuleers et al. (2016) want to encourage the development of such an alternative outside-in approach, it would be important to link this perspective to a well-defined research puzzle and relevant theoretical frames.

In their article, Keuleers et al. rely on the model of the policy cycle to make a distinction between the three approaches (Keuleers et al., 2016: 349–352). Journal articles about agenda-setting, policy-formulation and decision-making are coded as inward-looking. The articles dealing with the implementation of EU foreign policy fit into the inside-out approach. Finally, articles on the impact and evaluation of EU foreign policy are all about outside-in perspectives. Keuleers et al. (2016) therefore use the policy cycle as a structuring device to provide a snapshot of the EU foreign policy discipline. Yet this presents several challenges.

First, research agendas on EU foreign policy do not necessarily have much to do with the policy cycle. Research on actorness, normative power, Europeanisation and governance, for instance, concerns mostly the *structure and institutions* of EU foreign policy; not the *actual policy* made within the system. While structure and institutions often impact on policy and behaviour, they are traditionally seen as different loci of academic research. We would actually argue that the policy cycle – and foreign policy analysis more generally – is a distinct approach in itself, which has also been imported from a cognate field (White, 2001). It is thus unclear why the policy cycle would be a useful structuring device for an academic literature which goes beyond policy-making.

Second, the policy cycle is mainly a heuristic device, as the authors recognise themselves, ‘limited by its descriptive character and lack of explanatory power, which means it can never be the sole conceptual foundation for a research project’ (Keuleers et al., 2016: 348). The 17 example research questions, which Keuleers et al. (2016: 349–350) identify on the basis of the policy cycle, indeed primarily deal with the ‘what’ and the ‘how’ rather than the ‘why’. Such research questions lack explanatory power and are not a solid basis for a full-fledged new research agenda.

We embrace academic pluralism and value the exchange between the worlds of academia and policy. It is, however, doubtful whether academic research on EU foreign policy is best served by descriptive policy questions on impact and evaluation. As research and journal articles are (and should be) largely about building and testing theories, a theoretically-informed case for the outside-in perspective – next to empirical and normative considerations (Keuleers et al., 2016: 360) – is indispensable for the outside-in approach to become an attractive driver for new research projects.

## Towards future research agendas

Through their empirical analysis of a dataset of 451 articles, Keuleers et al. (2016) provide an interesting snapshot of the research agenda between 2010 and 2014. Against the background of the earlier mentioned three key drivers for academic research, the emphasis on inward-looking and inside-out questions is not surprising. By way of conclusion, we would like to briefly explore likely future research agendas.

Making predictions about the future is always risky business. Still if we continue to reason along the lines of Dunne et al. (2013), with their emphasis on the importance of the historical context for the academic research agenda, it is quite likely that the rapidly changing geopolitical environment and the new tensions in Europe will result in new puzzles. At a moment of increased international uncertainty, scholars may turn their attention to the implications of this evolving global environment, conceptualised as a multi-polar, multi-partner, multi-culture or even multi-order world (Flockhart, 2016; High Representative, 2015; Petito, 2016). This changing context may also give rise to renewed attention for grand theories, more appropriate to deal with macro-level questions.

Second, scholars will likely continue to import theories from cognate disciplines. The so-called practice turn (Adler and Pouliot, 2011), for instance, shows considerable promise (e.g. Bicchi and Bremberg, 2016). The debate on the EU democratic deficit has been broadened to research on the legitimacy of EU foreign policy (Sjursen, 2011). A further significant development is the increasing embedding of academic research on EU foreign policy in other related areas such as security studies, conflict studies, international political economy, and area studies: this points in a direction whereby EU foreign policy is less seen as *sui generis* and more as mainstream.

Finally, as a field grows more mature, we see a more systematic empirical testing of theories, new variables introduced and scope conditions defined. Following Mearsheimer and Walt (2013), we are cautious about the promise of this development. As noted above, there is a risk that empirical analyses become detached from theories.

## Notes

1. Robert Cooper (2005), a former advisor of Javier Solana used, for example, a variation in a speech.

2. Jørgensen (2015) also finds imbalances.
